# Preparation of Perovskite-Type LaMnO_3_ and Its Catalytic Degradation of Formaldehyde in Wastewater

**DOI:** 10.3390/molecules29163822

**Published:** 2024-08-12

**Authors:** Qingguo Ma, Pengcheng Huo, Kesong Wang, Ye Yuan, Songjiang Bai, Chentong Zhao, Wenzhuo Li

**Affiliations:** Department of Chemistry and Chemical Engineering, Taiyuan Institute of Technology, Taiyuan 030008, China; h19935488662@outlook.com (P.H.); wks15364929146@163.com (K.W.); 19934924885@vip.163.com (Y.Y.); 18734854908@163.com (S.B.); 19935347014@163.com (C.Z.); 17789196649@163.com (W.L.)

**Keywords:** formaldehyde, perovskite, catalytic oxidation, LaMnO_3_, degradation

## Abstract

Formaldehyde (HCHO) is identified as the most toxic chemical among 45 organic compounds found in industrial wastewater, posing significant harm to both the environment and human health. In this study, a novel approach utilizing the Lanthanum-manganese complex oxide (LaMnO_3_)/peroxymonosulfate (PMS) system was proposed for the effective removal of HCHO from wastewater. Perovskite-Type LaMnO_3_ was prepared by sol-gel method. The chemical compositions and morphology of LaMnO_3_ samples were analyzed through thermogravimetric analysis (TG), X-ray diffraction (XRD), X-ray photoelectron spectroscopy (XPS), and transmission electron microscopy (TEM). The effects of LaMnO_3_ dosage, PMS concentration, HCHO concentration, and initial pH on the HCHO removal rate were investigated. When the concentration of HCHO is less than 1.086 mg/mL (5 mL), the dosage of LaMnO_3_ is 0.06 g, and n(PMS)/n(HCHO) = 2.5, the removal rate of HCHO is more than 96% in the range of pH = 5–13 at 25 °C for 10 min. Compared with single-component MnO_2_, the perovskite structure of LaMnO_3_ is beneficial to the catalytic degradation of HCHO by PMS. It is an efficient Fenton-like oxidation process for treating wastewater containing HCHO. The LaMnO_3_ promoted the formation of SO_4_•^−^ and HO•, which sequentially oxidized HCHO to HCOOH and CO_2_.

## 1. Introduction

ABO_3_ perovskite oxides containing transition metals are widely used, especially Lanthanum-containing manganate (LaMnO_3_), which has attracted much attention due to its excellent performance in electrochemical [1,2,3,4,5] and catalytic reactions [6,7,8,9]. LaMnO_3_ is widely used in catalytic oxidation reactions because of its catalytic activity, chemical stability, and high oxygen adsorption capacity [10]. In the catalytic oxidation of toluene, the conversion rate of toluene is over 90% at 330 °C because of the abundant oxygen vacancy in LaMnO_3_ [11]. The catalyst performance and stability were tested in the oxidation of ethane; the experimental results showed that the conversion of ethane was promoted by the activation of C-H [12]. LaMnO_3_ has obvious catalytic activity in the conversion rate of d-glucose to malonic acid, lactic acid, and levoacylpropionic acid. In the best conditions, 69.5 mol.% of lactic and levulinic acid were obtained [13]. The surface defects of LaMnO_3_ were used to catalyze the oxidation of CO. The results showed that the O vacancy was conducive to the CO interaction, and the Mn vacancy was conducive to carbonate formation [14]. The band gap energies of LaMnO_3_ in full spectrum (UV-Vis-IR) absorption is 0.75 eV. After Fe-Ni doping, the UV absorption of La_0.95_Fe_0.025_Ni_0.025_MnO_3_ showed a blue shift and the degradation time of high concentration Congo red dye (40 ppm) was shortened; the degradation time was 45 min [15]. Peroxymonosulfate (PMS) was activated with LaMnO_3_ as a catalyst and the degradation rate of Ciprofloxacin antibiotics was 47.1% by Fenton-like catalytic oxidation. After the introduction of boron nitride quantum dots (BNQDs), the degradation of Ciprofloxacin antibiotics was significantly improved to 86.5% [16]. The catalytic activity of Ag-, Y-, and Pd-doped LaMnO_3_, and pure LaMnO_3_ were studied for the methyl orange removal. The results showed that the catalytic activity of Ag-doped LaMnO_3_ was slightly better than that of other catalysts. The maximum removal of methyl orange was 97% [17]. Undoped and Eu, Ho, Tb-doped LaMnO_3_ materials were synthesized and studied in the removal rate of 17α-ethynylestradiol from an aqueous environment. The best removal efficiency of 17α-ethinylestradiol was 77% after 30 min of UV irradiation in the presence of LMO: Ho [18]. Because of its special crystal structure, perovskite has great application potential in catalytic hydrogen production [19], electrocatalysis [20], catalytic synthesis [21], catalytic combustion [22], electromagnetic wave absorbing material [23], and so on.

Formaldehyde (HCHO) is identified as the most toxic chemical among 45 organic compounds found in industrial wastewater, posing significant harm to both the environment and human health [24]. HCHO is widely used in various industrial manufacturing because of its high reactivity, resulting in toxic and harmful HCHO wastewater [25,26,27,28]. A large amount of HCHO wastewater was produced in the leather industry [29], preparation of urea-HCHO resin [30,31], and Phenol-HCHO resin production [32,33]. There are many methods for removing HCHO, including physical, chemical, biochemical, photochemical, electro-chemical, and thermal methods [34,35,36]. Studies have shown that physical adsorption is less costly, more efficient, and easier to implement than other methods [37,38]. However, there is no chemical reaction to mineralize the HCHO; HCHO is just adsorbed into the material and will still pollute the environment if desorbed. The practical application of biochemical and photo-chemical methods is commonly high cost, time consuming, or energy intensive [39,40]. In particular, HCHO has a strong biological resistance and toxicity to microorganisms, seriously restricting the application of biological treatment [41].

Chemical oxidation, particularly Fenton oxidation, is widely employed in wastewater treatment. Fenton reaction involves the reaction of H_2_O_2_ with Fe^2+^ to produce ·OH radicals, which effectively oxidize pollutants in wastewater. However, the depletion of Fe^2+^ and H_2_O_2_ (Fe^2+^ is oxidized to Fe^3+^) leads to the termination of the reaction. So Fenton oxidation requires the addition of catalysts and oxidants, and will produce iron sludge [42,43]. To overcome this problem, Fenton-like oxidations have been extensively explored as a more efficient strategy for removing pollutants from wastewater. When HCHO is degraded by electro-Fenton, HCHO is distributed in the electrolyte, while the free radicals are only distributed on the cathode surface. In addition, the short survival time (10^−6^–10^−3^ s) [44] of free radicals in water leads to the useless consumption of free radicals, thus reducing the reaction efficiency [45]. The Photo-Fenton system is superior to the Fenton system in the removal rate and complete mineralization rate of HCHO, because the presence of light regenerates Fe^3+^ ions to Fe^2+^, which can then react with more H_2_O_2_ [42]. Although the Photo-Fenton process is very effective in the treatment of HCHO, the pH value of the degradation reaction is limited to a certain range, and ultraviolet radiation is required [46]. C. Gao innovatively applied PMS activation technology to remove HCHO and produce hydrogen, when the initial HCHO concentration was 0.722 mol L^−1^, the degradation rate of HCHO reached 30% [47]. J. Wei studied the degradation of nimesulide by CoTiO_3_/TiO_2_. The experimental results showed that the CoTiO_3_/TiO_2_ heterostructure promoted the targeted adsorption and activation of PMS, and the degradation rate of nimesulide was maintained above 91% [48]. In our previous experiments, we found that the complete removal of HCHO during degradation did not mean that HCHO was well treated because other contaminants, such as formic acid, were also produced [46,49]. Moussavi et al. used the catalytic advanced oxidation process and achieved 65.6% COD removal and 79% HCHO removal, but handling this technique was challenging [50].

It is important to obtain a catalyst that can simultaneously improve the ability of hydrogen peroxide or PMS, can be recycled, and has deactivation resistance for the oxidation of HCHO. This study investigates the efficiency of Fenton-like oxidation for treating wastewater containing HCHO. Two primary challenges must be addressed to facilitate the efficient degradation of HCHO through Fenton-like oxidation. Firstly, controlling the formation rate of free radical OH is imperative. Secondly, ensuring sufficient contact between free radicals and HCHO is essential. In other words, the catalyst can adsorb HCHO and oxidants, and can catalyze the oxidant to produce free radicals.

## 2. Results

### 2.1. Draw HCHO Standard Curve

**Figure 1:**
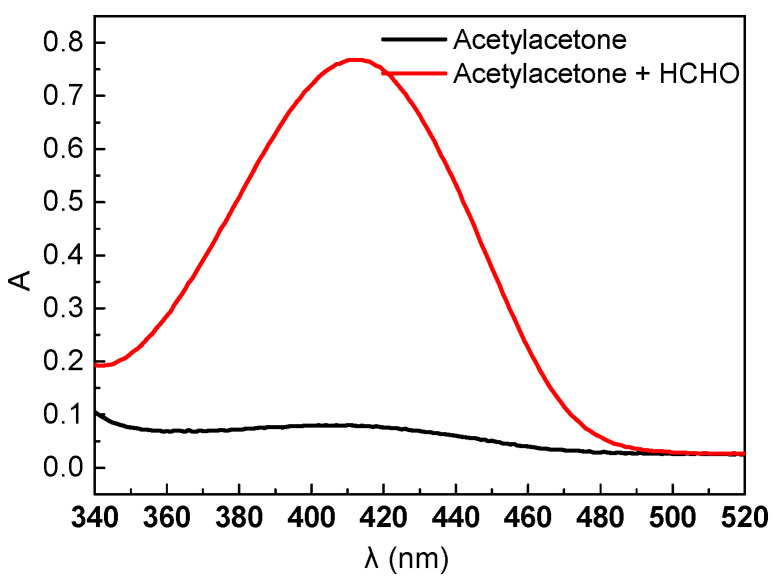
Wavelength scanning curve.

Add 1 mL of acetylacetone solution (0.5% V/V%) and 30 μL of HCHO solution (1.086 mg/mL) to a 10 mL colorimetric tube, then fill it up with deionized water up to the scale line, shake well, and react for 3 min at 100 °C. After cooling down, use a UV-visible photometer to determine that the maximum absorption wavelength of the HCHO-acetylacetone solution is 414 nm as depicted in Figure 1.

**Figure 2:**
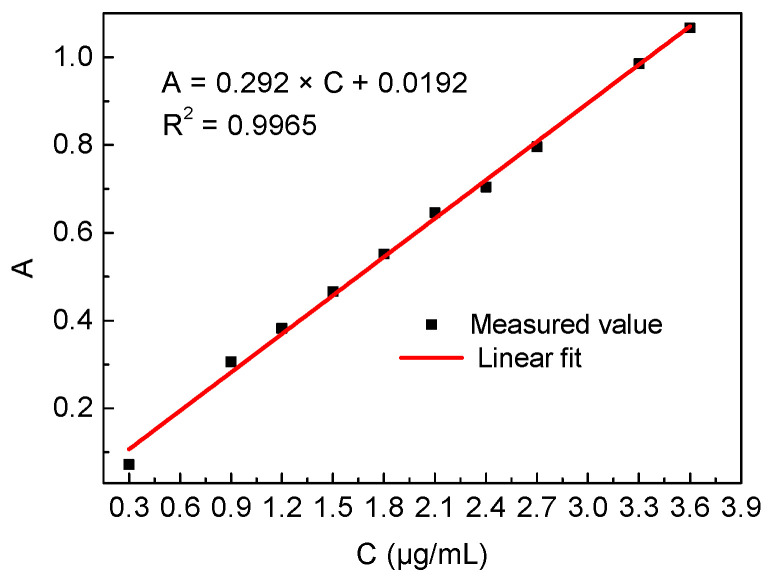
Standard curve of HCHO concentration and absorbance.

Figure 2 shows the standard curve of HCHO concentration and absorbance relationship. The linear fit within a range of 0.3–3 μg/mL was found to be 0.9966 for calculating HCHO concentration using this fitting curve.

### 2.2. Catalyst Characterization

**Figure 3:**
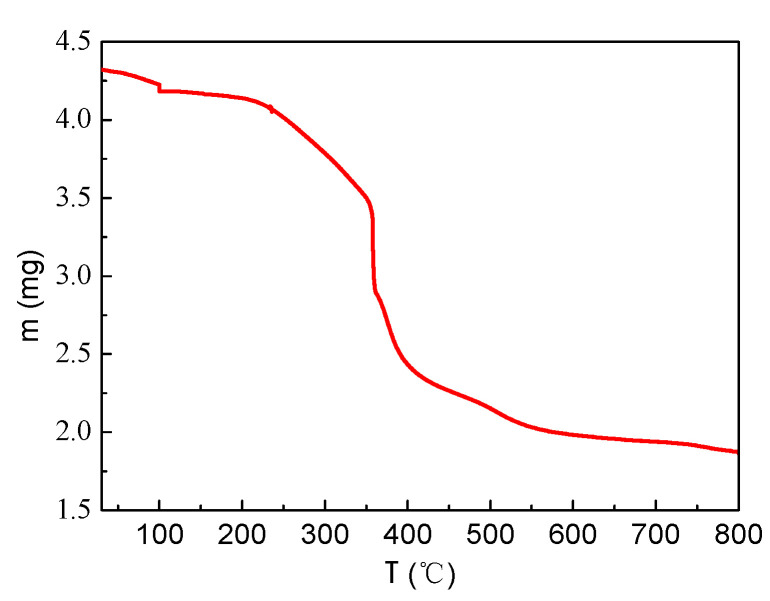
TG diagram of manganese-lanthanum citrate composite.

As shown in Figure 3, the weight loss of the sample is more obvious at 200~400 °C, which may be due to the decomposition of excess citric acid. At 400~600 °C, the loss of weight is reduced, which may be manganese-lanthanum citrate composite decomposed at this temperature. To ensure the conversion of manganese lanthanum citrate composite into oxide, the calcination temperature was set at 700 °C.

**Figure 4:**
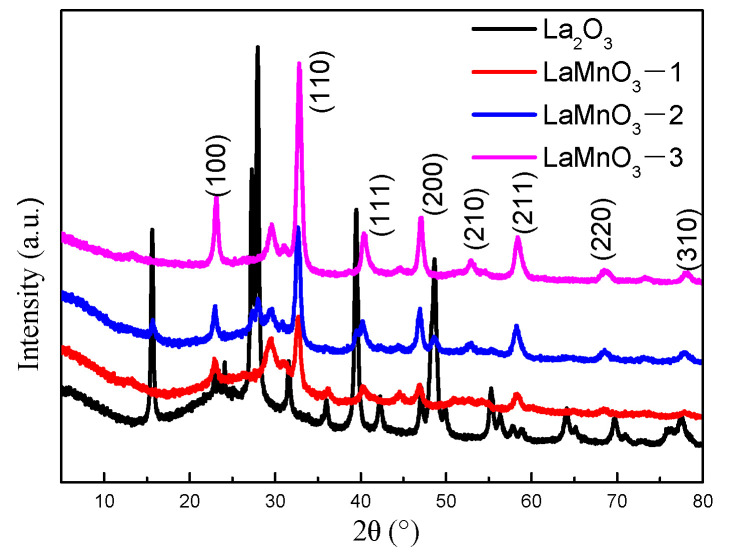
XRD patterns of Lanthanum-manganese complex oxides.

Peaks shown in Figure 4 (2θ = 22.88°, 32.76°, 40.2°, 46.92°, 52.7°, 58.1°, 68.6°, 77.8°) are in good agreement with the perovskite structure of LaMnO_3_ (JCPDS No: 89-8775) [19,51,52]. The peaks at 2θ = 29.4°, 44.2°, and 73.2° (LaMnO_3_-1, LaMnO_3_-2, LaMnO_3_-3 in Figure 4) may correspond to the crystallization peak of MnO_2_ [7]. In addition, since the phase La_2_MnO_4_ is only stable above 1650 K and at low O partial pressures [52], the presence of La_2_MnO_4_ is further ruled out. With the increase in lanthanum content, the crystal diffraction peak intensity of LaMnO_3_ increases, but the crystal peak of La_2_O_3_ does not appear. It is possible that La_2_O_3_ is highly dispersed in LaMnO_3_ and does not form crystals or does not appear because of its low diffraction intensity.

**Figure 5:**
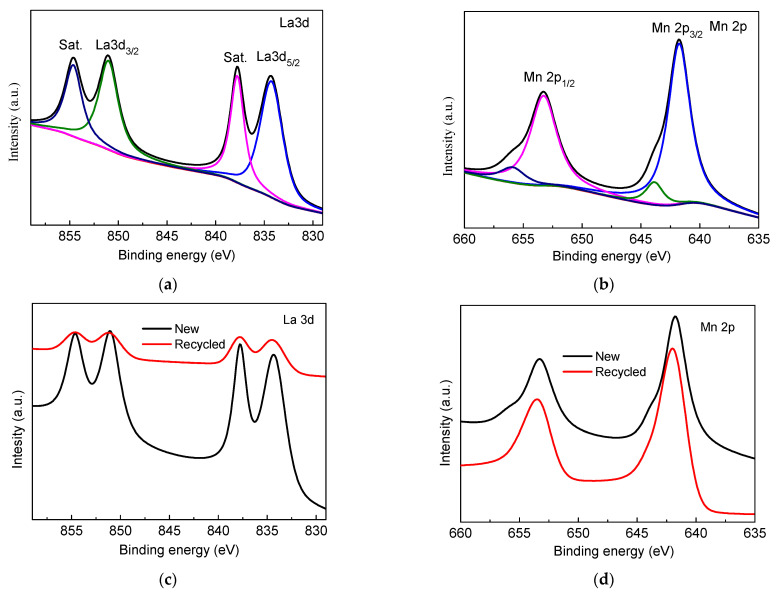
XPS of LaMnO_3_ ((**a**): La3d, (**b**): Mn2p, (**c**): La 3d in recycled LaMnO_3_-3, (**d**): Mn 3d in recycled LaMnO_3_-3)).

XPS of LaMnO_3_-3 is shown in Figure 5. The peaks at 851.0 and 854.7 eV are assigned to La3d_3/2_, and the peaks at 834.3 eV and 837.8 eV are assigned to La3d_5/2_. The typical peaks are in correspondence to La^3+^. The peaks at 641.9 eV (Mn 2p_3/2_) and 653.3 eV (Mn 2p_1/2_) correspond to Mn^3+^, while the two peaks at 643.9 eV and 656.0 eV are ascribed to Mn^4+^. In the lattice of LaMnO_3_, Mn^3+^ ions are the intrinsic valence of Mn, while Mn^4+^ stems from the existence of La cation vacancies [4]. The removal rates of HCHO decreased from 96.65% to 90.3% and the catalytic efficiency only decreased by 6.35% after the catalyst was reused three times. The structure of LaMnO_3_-3 before and after the catalytic oxidation reaction was characterized by XPS. As shown in Figure 5 the structure of LaMnO_3_-3 has not changed after the catalytic oxidation reaction. The results show that the LaMnO_3_ was stable.

**Figure 6:**
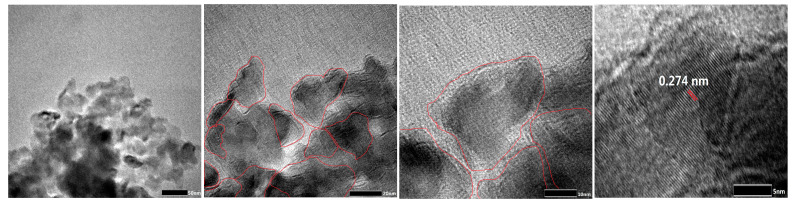
TEM images of LaMnO_3_.

The microstructure of LaMnO_3_-3 was revealed in detail by TEM analysis. As shown in Figure 6, the particle size of the sample was about 20–50 nm. The lattice fringes of LaMnO_3_-3 were obtained. The spacing of 0.274 nm was consistent with the (200) crystal plane in the X-ray diffraction spectra of the perovskite structure of LaMnO_3_ [53].

### 2.3. Catalytic Performance of LaMnO_3_ for Degradation of HCHO

**Figure 7:**
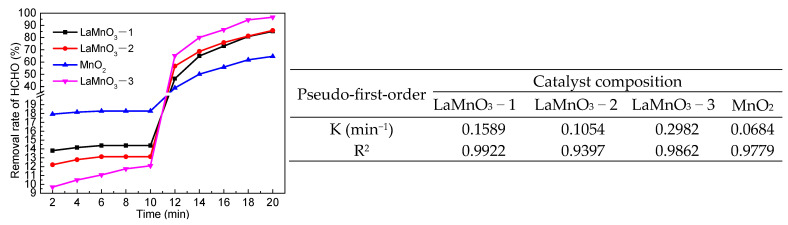
Effect of catalyst composition on the removal rate of HCHO (5 mL HCHO solution (1.086 mg/mL), catalyst 0.06 g, add PMS 0.13 g after 10 min, 25 °C).

**Figure 8:**
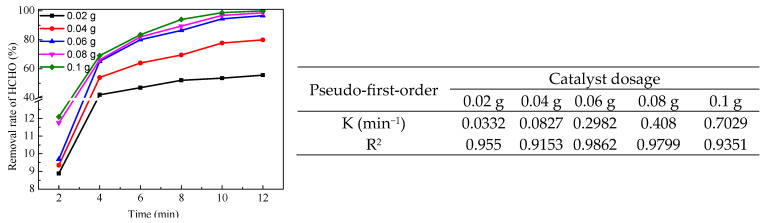
Effect of catalyst dosage on the removal rate of HCHO (5 mL HCHO solution (1.086 mg/mL), add PMS 0.13 g after 2 min, 25 °C).

The catalytic properties of LaMnO_3_ with different lanthanum contents on the degradation of HCHO were investigated. Figure 7 shows that the removal rate of HCHO increased with the increase in lanthanum content in the catalyst. The adsorption of the catalysts on HCHO is weak, and the catalyst with the best adsorption is MnO_2_. The maximum adsorption rate of HCHO within 10 min is 18.27%, and the adsorption rate changes little after 2 min with the extension of time, which indicates that the removal of HCHO is mainly through catalytic oxidation reaction. Under the condition of no catalyst, using PMS as an oxidant, the removal rate of HCHO was 28.5%. Using MnO_2_ as catalyst, the removal rate of HCHO was 64.69%, which was lower than the removal rate of formaldehyde using LaMnO_3_ as catalyst. The removal rates of HCHO were 38.2% and 20.55%, respectively, when the same amount of hydrogen peroxide was used instead of PMS as the oxidant and LaMnO_3_ or MnO_2_ as the catalyst to catalyze the degradation of HCHO. The potassium iodide-starch test paper test showed that MnO_2_ promoted the decomposition of hydrogen peroxide and reduced the oxidant in the reaction system. The same test method confirmed that LaMnO_3_ promoted the decomposition of hydrogen peroxide. Although LaMnO_3_ can also catalyze the decomposition of PMS, it is much slower than its catalytic decomposition of hydrogen peroxide. Hydrogen peroxide breaks down in 6 min, while PMS takes 90 min. The results indicate that the perovskite structure of LaMnO_3_ is beneficial to the catalytic degradation of HCHO by PMS. As can be seen from Figure 8 the removal rate of HCHO increased with the increase in catalyst dosage. However, when the amount of catalyst increased to 0.06 g, the removal rate of HCHO increased slightly with the increase in catalyst dosage. The results indicated that LaMnO_3_ catalyzes PMS to generate SO_4_•^−^ and SO_4_•^−^ was more stable than HO•. Therefore, increasing catalyst dosage does not affect the stability of SO_4_•^−^.

**Figure 9:**
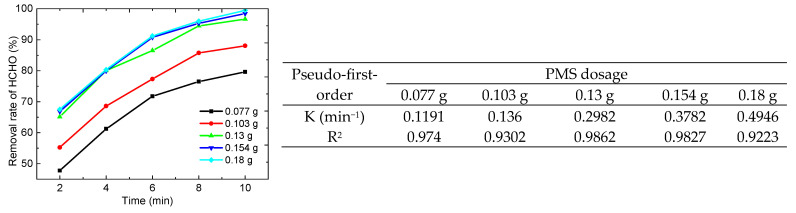
Effect of PMS dosage on the removal rate of HCHO (5 mL HCHO solution (1.086 mg/mL), catalyst 0.06 g, 25 °C).

The effect of different PMS dosages on the removal rate of HCHO was tested. As can be seen from Figure 9, the removal rate of HCHO increased with the increase in PMS dosage. However, when the amount of PMS increased to 0.13 g, the removal rate of HCHO increased slightly with the increase in PMS dosage. This is because increasing the amount of PMS without changing the amount of catalyst slightly changes the production of SO_4_•^−^.

**Figure 10:**
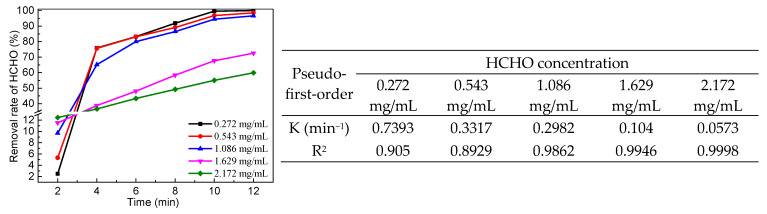
Effect of HCHO concentration on the removal rate of HCHO (n(PMS)/n(HCHO) = 2.5, catalyst 0.06 g, add PMS after 2 min, 25 °C).

As shown in Figure 10, when the concentration of HCHO was more than 1.629 mg/mL, the removal rate of HCHO was less than 80%. It is possible that the amount of SO_4_•^−^ produced under the same catalyst dosage may affect the degradation of HCHO. When the amount of catalyst was constant, the amount of SO_4_•^−^ produced was limited even if the amount of oxidant was increased. Therefore, when the concentration of HCHO was more than 1.629 mg/mL, the effects of the amount of catalyst and PMS on the conversion of HCHO need to be further investigated. The COD values of the HCHO solutions with different concentrations were measured by a water quality analyzer, and the COD removal rates were calculated. When the concentration of HCHO was 0.272 mg/mL, 0.543 mg/mL, 1.086 mg/mL, 1.629 mg/mL, and 2.172 mg/mL, the COD removal rates were 83.5%, 80.3%, 74.4%, 52.3%, and 40.8%, respectively. These results suggest that PMS cannot fully mineralize HCHO. The production of formic acid results in a low COD removal rate.

At pH 1, 3, 5, 7, 9, 11, and 13, the removal rate of HCHO was 76.06%, 93.72%, 99.39%, 96.65%, 97.77%, 99.5%, and 100%, respectively. The removal rate of formaldehyde is lower under acidic conditions. It may be that hydrogen ions hinder the formation of SO_4_•^−^ and •OH under acidic conditions. Therefore, fewer free radicals can participate in the degradation of formaldehyde under acidic conditions [54].

## 3. Discussion

When 1 mL of deionized water was added to the reaction system, the removal rate of HCHO was 98.65%, and when 1 mL of tert-butyl alcohol was added, the removal rate of formaldehyde decreased to 85.41%. Although the radical quenching agent of tert-butanol inhibited the degradation of HCHO, the decrease degradation rate of HCHO is not very large. The results show that LaMnO_3_ catalyzed PMS to generate SO_4_•^−^ and HO•, and SO_4_•^−^ was dominant.

The changes in the composition of the HCHO solution before and after the catalytic oxidation reaction were analyzed by HPLC. After the catalytic oxidation of HCHO, two peaks appeared, at 2.93 min and 3.17 min. When HCHO was replaced with water and the same amount of LaMnO_3_-3 and PMS were added, only one peak appeared at 2.93 min. The peak appeared at 3.17 min and was verified with a formic acid solution.

LaMnO_3_-3 calcined in air at 700 °C was further calcined at 900 °C and 1100 °C for 2 h, respectively. LaMnO_3_-3 calcined at 900 °C and 1100 °C were marked as LaMnO_3_-900 and LaMnO_3_-1100. The catalytic degradation of HCHO by LaMnO_3_-900 and LaMnO_3_-1100 was investigated (5 mL HCHO solution (1.086 mg/mL), catalyst 0.02 g, PMS 0.13 g, 25 °C, 10 min). The removal rates of HCHO were 43.77% and 42.39%. Under the same conditions, the removal rate of HCHO was 55.57 for the LaMnO_3_-3 calcined at 700 °C. These results suggest that with the increase in calcination temperature, the removal rate of HCHO decreased. We speculate that the charge of manganese changes at elevated temperatures [55,56]. Since Mn^4+^ is stabilized in the perovskite phase [52], the increase in calcination temperature leads to the conversion of Mn^3+^ to Mn^4+^. Under oxidizing conditions, the formation of OH is due to the charge change in Mn in LaMnO_3_, which changes from Mn^3+^ to Mn^4+^.

**Figure 11:**
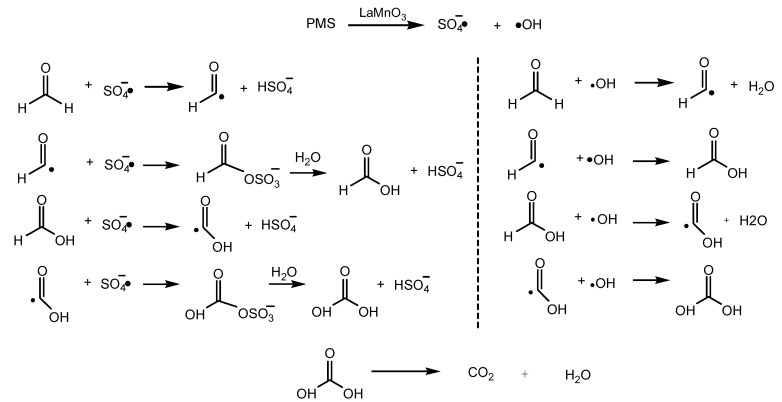
A possible reaction pathway for removing HCHO with PMS over LaMnO_3_.

Based on the above experimental results, a possible reaction pathway for removing HCHO with PMS over LaMnO_3_ was proposed. The reaction pathway was presented in Figure 11. The oxidation of HCHO is a Fenton-like oxidation reaction. The LaMnO_3_ promoted the formation of SO_4_•^−^ and HO•. HCHO was oxidized to HCOOH and CO_2_ by SO_4_•^−^ and HO•.

## 4. Materials and Methods

### 4.1. Preparation of HCHO Standard Solution

Add HCHO solution (approximately 1 mg/mL, 20 mL), sodium hydroxide solution (1 mol/L, 15 mL), and iodine solution (0.05 mol/L, 20 mL) to a iodine flask, shake well, and add sulfuric acid solution (0.05 mol/L, 20 mL) after 15 min. Then react for another 15 min, and titrate with sodium thiosulfate standard solution until the solution is pale yellow. Add starch indicator (1 mL) and continue titrating until the blue color is just faded. The volume of the sodium thiosulfate solution consumed is V_2_. Using 20 mL of deionized water instead of 20 mL of HCHO solution, titrated with standard sodium thiosulfate by the same method, the consumption volume is V_1_. The concentration of HCHO standard solution was calculated using Equation (1).

C = (V_1_ − V_2_) × C_Na_2_S_2_O_3__ × 15/20. (1)

Based on the volume of sodium thiosulfate standard solution consumed, the concentration of HCHO standard solution was 1.086 mg/mL.

### 4.2. Draw the HCHO Standard Curve

Next, add 0.3 mL, 0.6 mL, 0.9 mL, 1.2 mL, 1.5 mL, 1.8 mL, 2.1 mL, 2.4 mL, 2.7 mL, and 3.0 mL HCHO standard solution (10.86 μg/mL) to 10 colorimetric tubes (10 mL), then add deionized water to scale line. Shake well, and react for 3 min at 100 °C. After cooling, the photometer was used to measure the absorbance of the different concentrations of HCHO solution at 414 nm, and the standard curve of HCHO concentration-absorbance was drawn.

### 4.3. Preparation of Catalyst

Then 2.948 g of citric acid was added to 10 mL of deionized water and 3.567 g of lanthanum nitrate and 1.432 g of manganese nitrate solution (50%) were added to 10 mL of deionized water. The citric acid solution is then added to the mixed solution of lanthanum nitrate and manganese nitrate. The solution was stirred at 80 °C and evaporated into the gel. The gel was dried in an oven at 100 °C for 4 h, and then transferred to a muffle furnace calcined in air at 700 °C for 4 h then naturally cooled to room temperature. Then the sample materials with different ratios of lanthanum and manganese were prepared by the same process. The molar ratios of lanthanum nitrate to manganese nitrate were 1:1, 2:1, and 3:1, marked as LaMnO_3_-1, LaMnO_3_-2, and LaMnO_3_-3.

### 4.4. Catalyst Characterization

The thermal decomposition temperature of manganese-lanthanum citrate composite salt was determined by a thermogravimetric analyzer (TGA4000). The sample was determined in air with a flow rate of 20 mL/min. The temperature rising rate and range were 10 °C/min and 30~800 °C, respectively. The phase composition and crystal structure of the synthesized Lanthanum-manganese complex oxides (LaMnO_3_) were determined by X-ray diffraction analysis (XRD, Rigku, Smartlab, Tokyo, Japan), X-ray photoelectron spectrometer (XPS, ThermoFisher, ESCALAB 250Xi, Waltham, MA, USA), and the transmission electron microscope (TEM, JEOL, JEM-F200, Tokyo, Japan).

### 4.5. Catalytic Activity Experiment

The catalytic activity and stability of Lanthanum-manganese complex oxides were researched towards the degradation of HCHO with PMS as an oxidant. In a catalytic activity process, under stirring conditions, 0.06 g LaMnO_3_ was added into a 5 mL HCHO solution (1.086 mg/mL). Then, 0.13 g PMS (85 mM in the reaction solution) was added to the above mixture solution and kept at 25 °C for 10 min. Samples are taken from the mixture every 2 min during the reaction. The catalyst and PMS in the sample were removed by 0.45 μm filter membrane and sodium thiosulfate, respectively. At last, the sample solution was added to the acetylacetone solution to measure the absorbance and calculate the degradation rate of HCHO.

### 4.6. Analysis of Degradation Products

After the catalytic oxidation reaction (HCHO standard solution (5 mL), 0.06 g LaMnO_3_, 0.13 g PMS, 10 min), the degradation product was detected by an iChrom5000 high-performance liquid chromatography (HPLC) with diode array detector (DAD). The test conditions are as follows. Column: SinoChrom ODS-BP 5 um 4.6 × 150 mm, Column temperature: 30 °C, Mobile Phase: v (Methanol)/v (0.5% phosphoric acid solution) = (90/10), flow rate: 0.6 mL/min, Detection Wavelength: 210 nm.

In addition, the mixture of 5 mL water, 0.06 g LaMnO_3_, 0.13 g PMS, and HCOOH solution (11.2 mg/mL) was also detected by HPLC under the same test conditions.

### 4.7. Measurement of Chemical Oxygen Demand (COD)

The COD of the HCHO standard solution before and after the catalytic oxidation reaction was measured by a water quality analyzer (DGB-401). The test conditions are as follows. Take 2 mL of samples of HCHO standard solution (before and after the catalytic oxidation reaction) to a pre-configured COD detection reagent, cover the colorimetric tube tightly, shake and mix well. This solution was cooled after reacting at 150 °C for 2 h and tested for COD at 470 nm.

## 5. Conclusions

Lanthanum-manganese complex oxides were prepared by the sol-gel method. The perovskite structure was determined by XRD and TEM analysis, and the perovskite structure of LaMnO_3_ is beneficial to the catalytic degradation of HCHO by PMS compared with single-component MnO_2_. The catalytic activity of LaMnO_3_ was investigated for the removal of HCHO from the aqueous solution. It is an efficient Fenton-like oxidation process for the treatment of wastewater containing HCHO. The LaMnO_3_ promoted the formation of SO_4_•^−^ and HO•, which sequentially oxidized HCHO to HCOOH and CO_2_. Because the decomposition rate of H_2_O_2_ catalyzed by LaMnO_3_ is too fast and the HO• life is too short, it is not suitable for catalytic degradation of formaldehyde. The removal rate of HCHO increased with the increase in lanthanum content in the catalyst, catalyst dosage, and PMS dosage. The maximum removal of HCHO was 100% (5 mL HCHO solution (1.086 mg/mL), catalyst 0.1 g, PMS 0.13 g, 25 °C, 10 min). At pH 1, 3, 5, 7, 9, 11, and 13, the removal rate of HCHO was 76.06%, 93.72%, 99.39%, 96.65%, 97.77%, 99.5%, and 100%, respectively. The removal rate under acidic conditions is low because hydrogen ions hindered the formation of SO_4_•^−^ and •OH. When the concentration of HCHO is less than 1.086 mg/mL, and the amount of catalyst is constant, the removal rate of HCHO is more than 96%. However, when the concentration of HCHO is more than 1.629 mg/mL, the effects of the amount of catalyst and PMS on the conversion of HCHO need to be further investigated. The quenching tests proved that the removal of HCHO is through a radical pathway, and the key oxidant is SO_4_•^−^. The HO• life is too short to react with HCHO.

## Data Availability

No new data were created.
